# Supplementary material for: Comparative Transcriptome Analysis of Developing Seeds and Silique Wall Reveals Dynamic Transcription Networks for Effective Oil Production in Brassica napus L
Source: Int J Mol Sci. 2019 Apr 23;20(8):1982. doi: 10.3390/ijms20081982 (PMC6515390; doi:10.3390/ijms20081982)
Supplement: Supplementary file 1 [file ijms-20-01982-s001.zip › Supplementary Figures and Tables.docx]

**Supplementary Table S1.** Statistics of RNA-Seq reads and mapped reads.

| **Line** | Samples | Total raw reads | Total HQ clean reads | Total clean nucleotides basses (bp) | Q20 | GC (%) | Mapped reads to Bn genome sequence (%) | Uniquely mapped reads to Bn genome sequence (%) |
| --- | --- | --- | --- | --- | --- | --- | --- | --- |
| 1L99 (HOCL) | S-11D | 44,946,308 | 44,946,308 | 6,741,946,200 | 95.0 | 47.0 | 70.00 | 66.65 |
|  | S-16D | 41,462,656 | 41,462,656 | 6,219,398,400 | 94.5 | 47.1 | 69.62 | 66.17 |
|  | S-23D | 46,582,096 | 46,582,096 | 6,987,314,400 | 95.0 | 46.8 | 70.89 | 67.36 |
|  | S-30D | 47,676,912 | 47,676,912 | 7,151,536,800 | 95.1 | 47.3 | 71.53 | 67.56 |
|  | S-37D | 42,023,538 | 42,023,538 | 6,303,530,700 | 95.1 | 48.5 | 72.73 | 68.57 |
|  | S-44D | 51,902,498 | 51,902,498 | 7,785,374,700 | 94.8 | 48.1 | 71.69 | 62.92 |
|  | SW-11D | 49,612,280 | 49,612,280 | 7,441,842,000 | 95.1 | 47.2 | 76.13 | 67.48 |
|  | SW-16D | 43,489,302 | 43,489,302 | 6,523,395,300 | 95.0 | 47.0 | 70.49 | 66.99 |
|  | SW-23D | 52,166,620 | 52,166,620 | 7,824,993,000 | 94.9 | 47.1 | 71.27 | 67.77 |
|  | SW-30D | 47,165,248 | 47,165,248 | 7,074,787,200 | 94.8 | 47.1 | 71.11 | 67.52 |
|  | SW-37D | 54,746,282 | 54,746,282 | 8,211,942,300 | 95.1 | 46.7 | 70.56 | 67.06 |
|  | SW-44D | 42,815,514 | 42,815,514 | 6,422,327,100 | 94.7 | 46.6 | 70.27 | 66.81 |
|  | Total | 564,589,254 | 564,589,254 | 84,688,388,100 |  |  |  |  |
|  | Avg. | 47,049,105 | 47,049,105 | 7,057,365,675 | 94.9 | 47.2 | 71 | 67 |
| 1L363 (LOCL) | S-11D | 47,130,140 | 47,130,140 | 7,069,521,000 | 95.5 | 46.7 | 70.97 | 67.57 |
|  | S-16D | 43,079,466 | 43,079,466 | 6,461,919,900 | 95.3 | 47.1 | 71.48 | 67.58 |
|  | S-23D | 62,330,434 | 62,330,434 | 9,349,565,100 | 95. | 47.0 | 72.39 | 68.41 |
|  | S-30D | 64,213,788 | 64,213,788 | 9,632,068,200 | 95.7 | 47.1 | 71.92 | 66.45 |
|  | S-37D | 54,150,388 | 54,150,388 | 8,122,558,200 | 95.7 | 48.2 | 72.98 | 68.03 |
|  | S-44D | 59,481,446 | 59,481,446 | 8,922,216,900 | 95.7 | 48.0 | 72.83 | 68.55 |
|  | SW-11D | 57,647,608 | 57,647,608 | 8,647,141,200 | 95.7 | 47.1 | 72.84 | 69.04 |
|  | SW-16D | 46,730,486 | 46,730,486 | 7,009,572,900 | 95.4 | 47.2 | 71.88 | 68.32 |
|  | SW-23D | 53,610,584 | 53,610,584 | 8,041,587,600 | 95.7 | 47.5 | 72.94 | 69.23 |
|  | SW-30D | 51,641,370 | 51,641,370 | 7,746,205,500 | 95.5 | 47.1 | 72.42 | 68.51 |
|  | SW-37D | 56,723,798 | 56,723,798 | 8,508,569,700 | 95.7 | 47.6 | 72.43 | 68.81 |
|  | SW-44D | 54,118,944 | 54,118,944 | 8,117,841,600 | 95.9 | 47.4 | 72.08 | 68.47 |
|  | Total | 650,858,452 | 650,858,452 | 97,628,767,800 |  |  |  |  |
|  | Avg. | 54,238,204 | 54,238,204 | 8,135,730,650 | 95.6 | 47.3 | 72 | 68 |


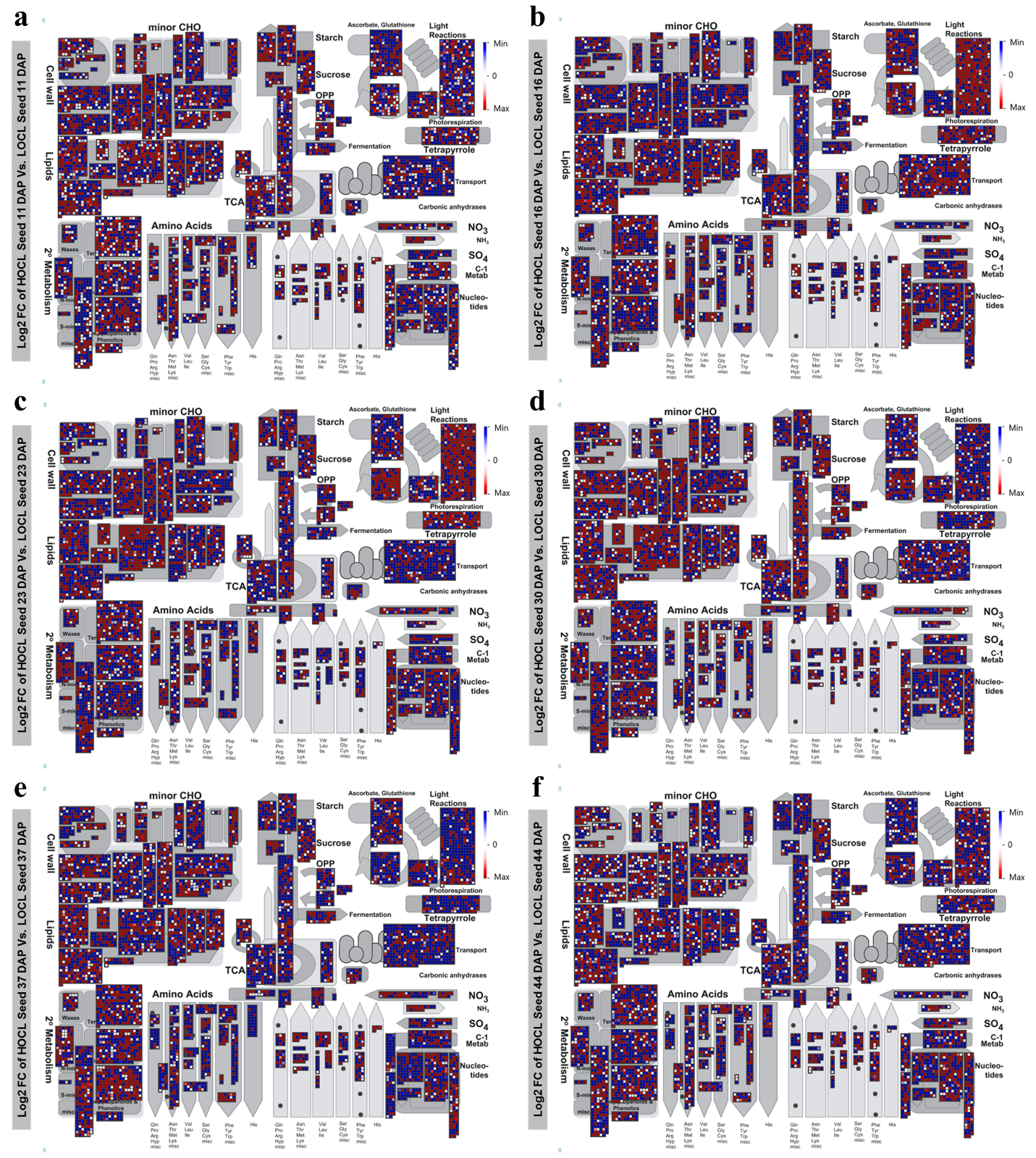


**Supplementary Figure S1.** An overview of genes expressed in seed tissue involved in major metabolism pathways at each developmental stage, revealed by MapMan. Total 6577 genes are visible in data points at each stage individually. Red dots designate genes expressed preferentially in HOCL, and blue expressed preferentially in LOC. (**a**) 11 DAP (**b**) 16 DAP (**c**) 23 DAP (**d**) 30 DAP (**e**) 37 DAP (**f**) 44 DAP.


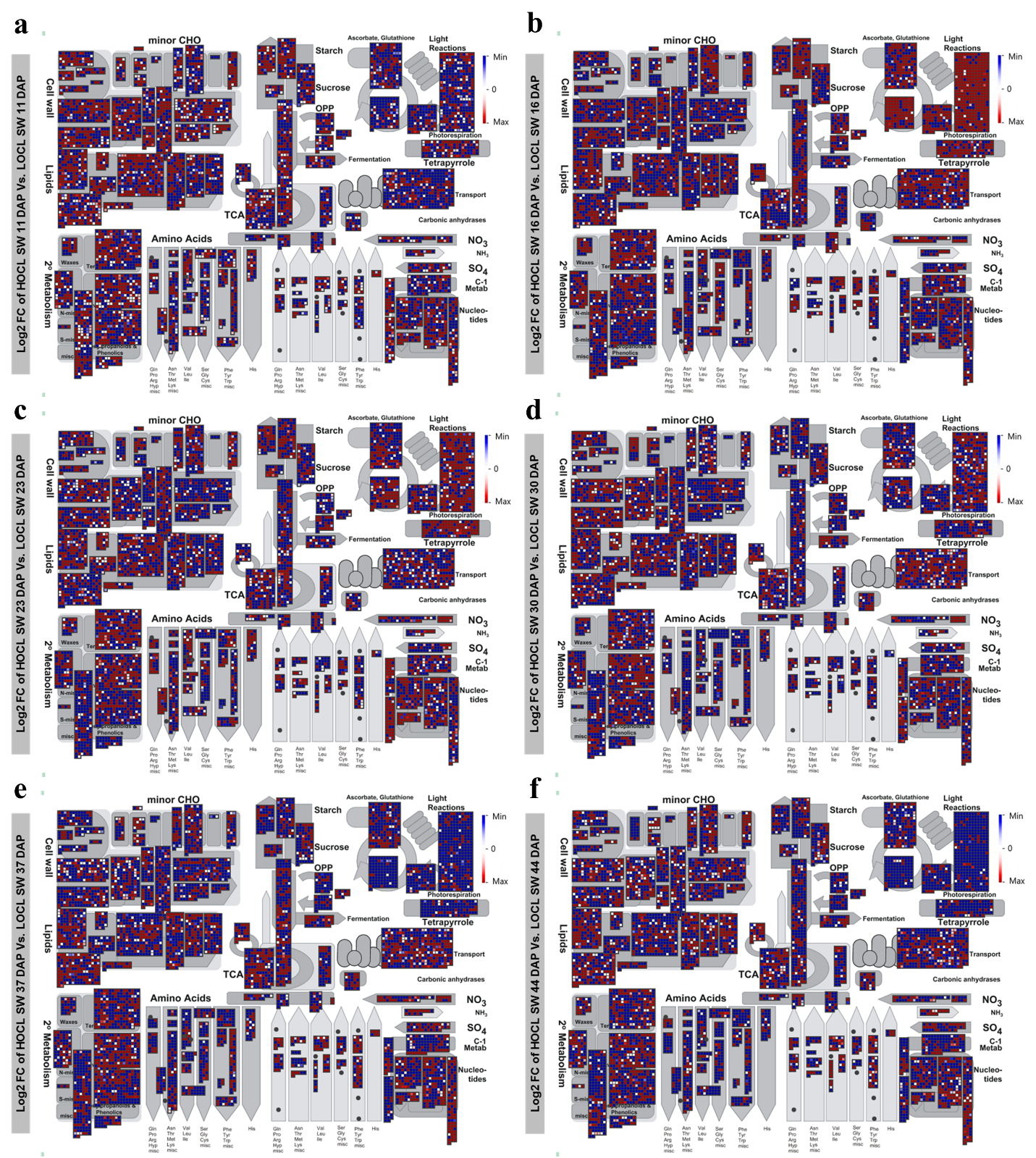


**Supplementary Figure S2.** An overview of genes expressed in SW tissue involved in major metabolism pathways at each developmental stage, revealed by MapMan. Total 6505 genes are visible in data points at each stage individually. Red dots designate genes expressed preferentially in HOCL, and blue expressed preferentially in LOC. (**a**) 11 DAP (**b**) 16 DAP (**c**) 23 DAP (**d**) 30 DAP (**e**) 37 DAP (**f**) 44 DAP.

**Supplementary Table S2.** A list of JA, ABA, and Auxin genes which are differentially expressed among HOCL and LOCL in seed and SW tissues.

| **Bn Gene ID** | **ATO** | **Description** | **Gene Name** | **Functional category** | **RTH** |
| --- | --- | --- | --- | --- | --- |
| BnaAnng01400D | AT5G05580 | Fatty acid desaturase 8 | *FAD8* | Hormone metabolic enzyme | JA |
| BnaA06g01390D | AT1G54040 | Epithiospecifier protein | *ESR* | Other signaling component | JA |
| BnaA09g02150D | AT3G27810 | Myb domain protein 21 | *MYB21* | Transcriptional factor | JA |
| BnaA04g02490D | AT3G57260 | Beta-1,3-glucanase 2 | *PR2* | Other signaling component | JA |
| BnaA04g02560D | AT3G56400 | WRKY DNA-binding protein 70 | *WRKY70* | Transcriptional factor | JA |
| BnaC05g14810D | AT1G19180 | Jasmonate-zim-domain protein 1 | *JAZ1* | Other signaling component | JA |
| BnaAnng10080D | AT1G54040 | Epithiospecifier protein | *ESR* | Other signaling component | JA |
| BnaA07g19600D | AT3G45140 | Lipoxygenase 2 | *LOX2* | Other signaling component | JA |
| BnaA01g23940D | AT3G23240 | Ethylene response factor 1 | *ERF1* | Transcriptional factor | JA |
| BnaC08g13270D | AT4G29010 | Enoyl-CoA hydratase/isomerase family | *AIM1* | Hormone metabolic enzyme | JA |
| BnaC06g18870D | AT3G45140 | Lipoxygenase 2 | *LOX2* | Other signaling component | JA |
| BnaA02g32350D | AT5G24770 | Vegetative storage protein 2 | *VSP2* | Other signaling component | JA |
| BnaC04g24330D | AT3G57260 | Beta-1,3-glucanase 2 | *PR2* | Other signaling component | JA |
| BnaAnng23590D | AT3G57260 | Beta-1,3-glucanase 2 | *PR2* | Other signaling component | JA |
| BnaC08g28160D | AT3G57260 | Beta-1,3-glucanase 2 | *PR2* | Other signaling component | JA |
| BnaC02g31070D | AT1G54040 | Epithiospecifier protein | *ESR* | Other signaling component | JA |
| BnaC01g34620D | AT3G17860 | Jasmonate-zim-domain protein 3 | *JAI3* | Other signaling component | JA |
| BnaC02g41060D | AT5G24770 | Vegetative storage protein 2 | *VSP2* | Other signaling component | JA |
| BnaC02g00870D | AT5G64310 | Arabinogalactan protein 1 | *AGP1* | Other signaling component | ABA |
| BnaC02g00990D | AT5G08790 | NAC (No Apical Meristem) domain transcriptional regulator superfamily protein | *anac081* | Other signaling component | ABA |
| BnaC02g45160D | AT5G66400 | Dehydrin family protein | *RAB18* | Other signaling component | ABA |
| BnaC02g02300D | AT5G05410 | DRE-binding protein 2A | *DREB2A* | Transcriptional factor | ABA |
| BnaC02g02310D | AT5G05410 | DRE-binding protein 2A | *DREB2A* | Transcriptional factor | ABA |
| BnaA01g03110D | AT4G33950 | Protein kinase superfamily protein | *OST1* | Kinase | ABA |
| BnaC09g03940D | AT5G25610 | BURP domain-containing protein | *RD22* | Other signaling component | ABA |
| BnaAnng05950D | AT5G08790 | NAC (No Apical Meristem) domain transcriptional regulator superfamily protein | *anac081* | Other signaling component | ABA |
| BnaC09g19020D | AT5G52300 | CAP160 protein | *RD29B* | Other signaling component | ABA |
| BnaA03g36320D | AT4G15560 | Deoxyxylulose-5-phosphate synthase | *CLA1* | Hormone metabolic enzyme | ABA |
| BnaA05g24050D | AT3G15500 | NAC domain containing protein 3 | *ANAC055* | Other signaling component | ABA |
| BnaA09g26450D | AT1G30100 | Nine-cis-epoxycarotenoid dioxygenase 5 | *NCED5* | Hormone metabolic enzyme | ABA |
| BnaA05g32630D | AT3G03450 | RGA-like 2 | *RGL2* | Transcriptional factor | ABA |
| BnaA05g32640D | AT3G03450 | RGA-like 2 | *RGL2* | Transcriptional factor | ABA |
| BnaC07g18360D | AT5G45340 | Cytochrome P450, family 707, subfamily A, polypeptide 3 | *CYP707A3* | Hormone metabolic enzyme | ABA |
| BnaC06g25430D | AT1G69260 | ABI five binding protein | *AFP1* | Other signaling component | ABA |
| BnaA09g48410D | AT1G09570 | Phytochrome A | *PHYA* | Kinase | ABA |
| BnaA09g51430D | AT1G02280 | Translocon at the outer envelope membrane of chloroplasts 33 | *PPI1* | Other signaling component | ABA |
| BnaC05g38150D | AT3G15500 | NAC domain containing protein 3 | *ANAC055* | Other signaling component | ABA |
| BnaC05g39180D | AT3G14440 | Nine-cis-epoxycarotenoid dioxygenase 3 | *NCED3* | Hormone metabolic enzyme | ABA |
| BnaC01g39470D | AT3G08550 | Elongation defective 1 protein / ELD1 protein | *ABI8* | Other signaling component | ABA |
| BnaC07g45200D | AT4G34710 | Arginine decarboxylase 2 | *ADC2* | Hormone metabolic enzyme | ABA |
| BnaCnng46690D | AT4G34890 | Xanthine dehydrogenase 1 | *XDH1* | Hormone metabolic enzyme | ABA |
| BnaC09g00020D | AT4G02570 | Cullin 1 | *AtCUL1* | Ubiquiting relative | Auxin |
| BnaA01g34610D | AT4G39950 | Cytochrome P450, family 79, subfamily B, polypeptide 2 | *CYP79B2* | Hormone metabolic enzyme | Auxin |
| BnaA09g00890D | AT4G02570 | Cullin 1 | *AtCUL1* | Ubiquiting relative | Auxin |
| BnaC09g01920D | AT3G28860 | ATP binding cassette subfamily B19 | *MDR1* | Transportor | Auxin |
| BnaA06g38980D | AT3G44300 | Nitrilase 2 | *NIT2* | Hormone metabolic enzyme | Auxin |
| BnaA09g02390D | AT3G28860 | ATP binding cassette subfamily B19 | *MDR1* | Transportor | Auxin |
| BnaCnng01290D | AT5G13930 | Chalcone and stilbene synthase family protein | *AtCHS* | Transportor | Auxin |
| BnaA08g04520D | AT4G31500 | Cytochrome P450, family 83, subfamily B, polypeptide 1 | *CYP83B1* | Hormone metabolic enzyme | Auxin |
| BnaA03g10890D | AT3G44300 | Nitrilase 2 | *NIT2* | Hormone metabolic enzyme | Auxin |
| BnaA06g14090D | AT5G62000 | Auxin response factor 2 | *ARF2* | Transcriptional factor | Auxin |
| BnaC08g05690D | AT4G31500 | Cytochrome P450, family 83, subfamily B, polypeptide 1 | *CYP83B1* | Hormone metabolic enzyme | Auxin |
| BnaA05g14370D | AT5G62000 | Auxin response factor 2 | *ARF2* | Transcriptional factor | Auxin |
| BnaA08g19440D | AT1G25490 | ARM repeat superfamily protein | *EER1* | Transportor | Auxin |
| BnaC08g12460D | AT1G05180 | NAD(P)-binding Rossmann-fold superfamily protein | *AXR1* | Ubiquiting relative | Auxin |
| BnaC03g54910D | AT3G44300 | Nitrilase 2 | *NIT2* | Hormone metabolic enzyme | Auxin |

ATO; *Arabidopsis thaliana* ortholog , RTH; Response to hormone.

**Supplementary Table S3.** A list of 75 ALM genes found differentially expressed among HOCL and LOCL.

| **Bn Gene ID** | **ATO** | **Description** | **Pathway** | **Gene Name** |
| --- | --- | --- | --- | --- |
| BnaA05g33110D | AT3G04290 | Li-tolerant lipase 1 | Cutin Synthesis & Transport 1 | *LTL1 / CD1* |
| BnaA07g22620D | AT1G73600 | S-adenosyl-L-methionine-dependent methyltransferases superfamily protein | Eukaryotic Phospholipid Synthesis & Editing | *PEAMT* |
| BnaA07g34320D | AT1G78690 | Phospholipid/glycerol acyltransferase family protein | Eukaryotic Phospholipid Synthesis & Editing | *LPLAT* |
| BnaA04g12720D | AT2G22240 | Myo-inositol-1-phosphate synthase 2 | Eukaryotic Phospholipid Synthesis & Editing | *MIPS2* |
| BnaA05g32880D | AT3G03520 | Non-specific phospholipase C3 | N/A | *NPC3 (ns)* |
| BnaA10g27550D | AT5G01220 | Sulfoquinovosyl diacylglycerol 2 | Prokaryotic Galactolipid, Sulfolipid, & Phospholipid Synthesis 2;Eukaryotic Galactolipid & Sulfolipid Synthesis | *SQD2* |
| BnaAnng01400D | AT5G05580 | FA desaturase 8 | Prokaryotic Galactolipid, Sulfolipid, & Phospholipid Synthesis 2;Eukaryotic Galactolipid & Sulfolipid Synthesis | *FAD8* |
| BnaA02g00400D | AT5G10170 | Myo-inositol-1-phosphate synthase 3 | Eukaryotic Phospholipid Synthesis & Editing | *MIPS3* |
| BnaA09g51530D | AT1G01120 | 3-ketoacyl-CoA synthase 1 | FA Elongation & Wax Biosynthesis | *KCS1* |
| BnaA02g15770D | AT1G72110 | O-acyltransferase (WSD1-like) family protein | FA Elongation & Wax Biosynthesis | *WSD* |
| BnaA07g31060D | AT1G73890 | Bi-functional inhibitor/lipid-transfer protein/seed storage 2S albumin superfamily protein | FA Elongation & Wax Biosynthesis | *LTP 5* |
| BnaA04g26960D | AT2G47240 | AMP-dependent synthetase and ligase family protein | FA Elongation & Wax Biosynthesis | *LACS1* |
| BnaA07g06180D | AT3G23840 | HXXXD-type acyl-transferase family protein | FA Elongation & Wax Biosynthesis | *CER2* |
| BnaA10g24560D | AT5G05960 | Bi-functional inhibitor/lipid-transfer protein/seed storage 2S albumin superfamily protein | FA Elongation & Wax Biosynthesis | *LTP 4* |
| BnaA10g19780D | AT5G13580 | ABC-2 type transporter family protein | FA Elongation & Wax Biosynthesis | *WBC6 / ABCG6* |
| BnaAnng29910D | AT5G55450 | Bi-functional inhibitor/lipid-transfer protein/seed storage 2S albumin superfamily protein | FA Elongation & Wax Biosynthesis | *LTP3* |
| BnaA05g33500D | AT3G02610 | Plant stearoyl-acyl-carrier-protein desaturase family protein | N/A | *DES2* |
| BnaA09g51510D | AT1G01090 | Pyruvate dehydrogenase E1 alpha | FA Synthesis | *PDH (E1 alpha)* |
| BnaA02g24400D | AT5G46290 | 3-ketoacyl-acyl carrier protein synthase I | FA Synthesis | *KASI* |
| BnaA10g26340D | AT5G03770 | KDO transferase A | Mitochondrial Lipopolysaccharide Synthesis | *AtKdtA* |
| BnaA03g41900D | AT4G15440 | Hydroperoxide lyase 1 | Oxylipin Metabolism 1 | *HPL* |
| BnaA01g12060D | AT4G22240 | Plastid-lipid associated protein PAP / fibrillin family protein | Pathway, function or subcellular location uncertain | *LP* |
| BnaA10g23290D | AT5G08030 | PLC-like phosphodiesterases superfamily protein | Pathway, function or subcellular location uncertain | *GPDEPDE* |
| BnaA09g40500D | AT2G26420 | 1-phosphatidylinositol-4-phosphate 5-kinase 3 | Phospholipid Signalling | *PIPK-IB* |
| BnaA07g22900D | AT1G72970 | Glucose-methanol-choline (GMC) oxidoreductase family protein | Cutin Synthesis & Transport 1 | *HTD* |
| BnaAnng19780D | AT4G36480 | Long-chain base1 | Sphingolipid Biosynthesis 1 | *LCB1* |
| BnaA10g15860D | AT5G19200 | NAD(P)-binding Rossmann-fold superfamily protein | Sphingolipid Biosynthesis 1 | *TSC10B* |
| BnaA07g26750D | AT1G67980 | Caffeoyl-CoA 3-O-methyltransferase | N/A | *N/A* |
| BnaA02g11130D | AT5G51950 | Glucose-methanol-choline (GMC) oxidoreductase family protein | Suberin Synthesis & Transport 1 | *N/A* |
| BnaAnng17300D | AT5G58860 | Cytochrome P450, family 86, subfamily A, polypeptide 1 | Suberin Synthesis & Transport 1 | *CYP86A1(Arabidopsis) / CYP86A33(potato)* |
| BnaA03g18600D | AT2G39420 | Alpha/beta-Hydrolases superfamily protein | Triacylglycerol & Fatty Acid Degradation | *MAGL* |
| BnaA05g31340D | AT3G05970 | Long-chain acyl-CoA synthetase 6 | Triacylglycerol & Fatty Acid Degradation | *LACS6* |
| BnaA05g25110D | AT3G14360 | Alpha/beta-Hydrolases superfamily protein | Triacylglycerol & Fatty Acid Degradation | *TAGL LAH* |
| BnaA01g14480D | AT4G25140 | Oleosin 1 | TAG Biosynthesis | *OBO* |
| BnaA01g19390D | AT3G51520 | Diacylglycerol acyltransferase family | TAG Biosynthesis | *DGAT2* |
| BnaAnng06440D | AT5G07560 | Glycine-rich protein 20 | TAG Biosynthesis | *PO* |
| BnaC05g47350D | AT3G04290 | Li-tolerant lipase 1 | Cutin Synthesis & Transport 1 | *LTL1 / CD1* |
| BnaC04g45690D | AT2G38110 | Glycerol-3-phosphate acyltransferase 6 | Cutin Synthesis & Transport 1 | *sn-2-GPAT6* |
| BnaC06g15590D | AT3G55030 | Phosphatidyl glycerol phosphate synthase 2 | Eukaryotic Phospholipid Synthesis & Editing | *PGPS / PGP2 KCS5 / CER60* |
| BnaC06g40070D | AT1G80950 | Phospholipid/glycerol acyltransferase family protein | Eukaryotic Phospholipid Synthesis & Editing | *LPEAT1* |
| BnaC05g48240D | AT3G02600 | Lipid phosphate phosphatase 3 | Prokaryotic Galactolipid, Sulfolipid, & Phospholipid Synthesis 1;Eukaryotic Galactolipid & Sulfolipid Synthesis;Phospholipid Signalling | *PP; AtLPP3* |
| BnaC05g00780D | AT1G01120 | 3-ketoacyl-CoA synthase 1 | FA Elongation & Wax Biosynthesis | *KCS1* |
| BnaC08g34530D | AT2G23180 | Cytochrome P450, family 96, subfamily A, polypeptide 1 | FA Elongation & Wax Biosynthesis | *CYP96A1* |
| BnaC04g07620D | AT2G37870 | Bi-functional inhibitor/lipid-transfer protein/seed storage 2S albumin superfamily protein | FA Elongation & Wax Biosynthesis | *(LTP5)* |
| BnaC03g21770D | AT2G38995 | O-acyltransferase (WSD1-like) family protein | FA Elongation & Wax Biosynthesis | *WSD* |
| BnaC04g50990D | AT2G48140 | Bi-functional inhibitor/lipid-transfer protein/seed storage 2S albumin superfamily protein | FA Elongation & Wax Biosynthesis | *EDA4 (LTP5)* |
| BnaC08g22920D | AT3G51600 | Lipid transfer protein 5 | FA Elongation & Wax Biosynthesis | *LTP1)* |
| BnaC01g19560D | AT4G27420 | ABC-2 type transporter family protein | FA Elongation & Wax Biosynthesis | *WBC9 / ABCG9* |
| BnaC02g01960D | AT5G06530 | ABC-2 type transporter family protein | FA Elongation & Wax Biosynthesis | *WBC23 / ABCG22* |
| BnaC02g00930D | AT5G64080 | Bi-functional inhibitor/lipid-transfer protein/seed storage 2S albumin superfamily protein | FA Elongation & Wax Biosynthesis | *(LTP5)* |
| BnaC03g04350D | AT5G10480 | Protein-tyrosine phosphatase-like, PTPLA | FA Elongation & Wax Biosynthesis | *HCD / PAS2* |
| BnaCnng20100D | AT5G11090 | serine-rich protein-related | FA Elongation & Wax Biosynthesis | *N/A* |
| BnaC05g48250D | AT3G02610 | Plant stearoyl-acyl-carrier-protein desaturase family protein | N/A | *DES2* |
| BnaCnng78040D | N/A | N/A | N/A | *N/A* |
| BnaC02g01120D | AT5G08415 | Radical SAM superfamily protein | FA Synthesis | *LS* |
| BnaC02g00470D | AT5G10160 | Thioesterase superfamily protein | FA Synthesis | *HAD* |
| BnaC03g04180D | AT5G10160 | Thioesterase superfamily protein | FA Synthesis | *HAD* |
| BnaC02g32230D | AT5G46290 | 3-ketoacyl-acyl carrier protein synthase I | FA Synthesis | *KASI* |
| BnaC05g42730D | AT3G10370 | FAD-dependent oxidoreductase family protein | Mitochondrial Phospholipid Synthesis | *SDP6* |
| BnaC08g37760D | AT1G17420 | Lipoxygenase 3 | Oxylipin Metabolism 1; Oxylipin Metabolism 2 | *LOX* |
| BnaC05g47870D | AT3G03310 | Lecithin: cholesterol acyltransferase 3 | Pathway, function or subcellular location uncertain | *PLAT* |
| BnaC02g22330D | AT1G73680 | Alpha dioxygenase | Oxylipin Metabolism 2 | *DOP* |
| BnaC09g52550D | AT3G25780 | Allene oxide cyclase 3 | Oxylipin Metabolism 1; Oxylipin Metabolism 2 | *AOX* |
| BnaC09g02340D | AT5G48370 | Thioesterase/thiol ester dehydrase-isomerase superfamily protein | Pathway, function or subcellular location uncertain | *ACT* |
| BnaC03g69220D | AT1G52570 | Phospholipase D alpha 2 | Phospholipid Signalling | *PLD &alpha* |
| BnaC03g73050D | AT4G11850 | Phospholipase D gamma 1 | Phospholipid Signalling | *PLD &gamma* |
| BnaC05g45860D | AT3G05970 | Long-chain acyl-CoA synthetase 6 | TAG & FA Degradation | *LACS6* |
| BnaC04g47480D | AT2G40890 | Cytochrome P450, family 98, subfamily A, polypeptide 3 | Suberin Synthesis & Transport 2 | *CYP98A3* |
| BnaC02g41740D | AT5G23190 | Cytochrome P450, family 86, subfamily B, polypeptide 1 | Suberin Synthesis & Transport 1 | *CYP86B1* |
| BnaC02g15440D | AT5G51950 | Glucose-methanol-choline (GMC) oxidoreductase family protein | Suberin Synthesis & Transport 1 | *N/A* |
| BnaC05g39290D | AT3G14360 | Alpha/beta-Hydrolases superfamily protein | TAG & FA Degradation | *TAGL LAH* |
| BnaC08g13270D | AT4G29010 | Enoyl-CoA hydratase/isomerase family | TAG & FA Degradation | *AIM1* |
| BnaC02g02780D | AT5G04040 | Patatin-like phospholipase family protein | TAG & FA Degradation | *SDP1* |
| BnaCnng57830D | AT5G50700 | Hydroxysteroid dehydrogenase 1 | TAG Biosynthesis | *STERO OBO or OBP* |
| BnaC09g27370D | AT5G51210 | Oleosin3 | TAG Biosynthesis | *OBO* |

ATO; Arabidopsis thaliana ortholog.

**Supplementary Table S4** A list of 42 Transcription factors (TFs) genes found differentially expressed among HOCL and LOCL.

| **Bn Gene ID** | **ATO** | **Description** | **Family** | **Symbol** |
| --- | --- | --- | --- | --- |
| BnaC05g23480D | AT1G30500 | Nuclear factor Y, subunit A7 | NF-YA | *NF-YA7* |
| BnaA05g17950D | AT1G32770 | NAC domain containing protein 12 | NAC | *NAC-SND1* |
| BnaA01g21580D | AT1G58110 | Basic-leucine zipper (bZIP) transcription factor family protein | bZIP | *bZIP-TF* |
| BnaA04g28800D | AT2G31220 | Basic helix-loop-helix (bHLH) DNA-binding superfamily protein | bHLH | *BHLH10* |
| BnaA05g32640D | AT3G03450 | RGA-like 2 | GRAS | *RGL-like2* |
| BnaC01g40250D | AT3G04070 | NAC domain containing protein 47 | NAC | *NAC-NAM* |
| BnaC01g44080D | AT3G16870 | GATA transcription factor 17 | GATA | *GATA-ZF TF* |
| BnaC01g33010D | AT3G19860 | Basic helix-loop-helix (bHLH) DNA-binding superfamily protein | bHLH | *bHLH121* |
| BnaC07g49020D | AT3G27010 | TEOSINTE BRANCHED 1, cycloidea, PCF (TCP)-domain family protein 20 | TCP | *TCP20* |
| BnaCnng20400D | AT3G56850 | ABA-responsive element binding protein 3 | bZIP | *ABA-RP* |
| BnaC03g26990D | AT4G00050 | Basic helix-loop-helix (bHLH) DNA-binding superfamily protein | bHLH | *bHLH* |
| BnaCnng68660D | AT4G00480 | Basic helix-loop-helix (bHLH) DNA-binding superfamily protein | bHLH | *MYC1* |
| BnaCnng32870D | AT4G02590 | Basic helix-loop-helix (bHLH) DNA-binding superfamily protein | bHLH | *UNE12* |
| BnaC09g00090D | AT4G02640 | bZIP transcription factor family protein | bZIP | *bZIP-BZO2H1* |
| BnaC08g11750D | AT4G24660 | Homeobox protein 22 | ZF-HD | *ZF-HD* |
| BnaAnng24640D | AT4G26150 | Cytokinin-responsive gata factor 1 | GATA | *GATA-TF* |
| BnaA03g53460D | AT4G36540 | BR enhanced expression 2 | bHLH | *BR EE2* |
| BnaC02g01720D | AT5G07100 | WRKY DNA-binding protein 26 | WRKY | *WRKY 26.1* |
| BnaC03g65280D | AT5G15210 | Homeobox protein 30 | ZF-HD | *HB29-like* |
| BnaC09g18980D | AT5G45050 | Disease resistance protein (TIR-NBS-LRR class) | WRKY | *TIR-NBS-LRR* |
| BnaA06g35350D | AT5G47660 | Homeodomain-like superfamily protein | Trihelix | *DNA-BP* |
| BnaA06g35340D | AT5G47670 | Nuclear factor Y, subunit B6 | NF-YB | *NF-YB6A* |
| BnaA10g05960D | AT5G50820 | NAC domain containing protein 97 | NAC | *NAC* |
| BnaA02g08430D | AT5G56960 | Basic helix-loop-helix (bHLH) DNA-binding family protein | bHLH | *bHLH041* |
| BnaA05g14370D | AT5G62000 | Auxin response factor 2 | ARF | *mtg10_20* |
| BnaC02g00280D | AT5G10510 | AINTEGUMENTA-like 6 | AP2 | *117M18_31* |
| BnaA01g23940D | AT3G23240 | Ethylene response factor 1 | ERF | *EREBP* |
| BnaC05g47270D | AT3G04450 | Homeodomain-like superfamily protein | G2-like | *HD-like* |
| BnaA09g03540D | AT5G29000 | Homeodomain-like superfamily protein | G2-like | *PHR1-LIKE 1* |
| BnaAnng28250D | AT5G51860 | K-box region and MADS-box transcription factor family protein | MIKC_MADS | *MADS-box* |
| BnaCnng20070D | AT5G11060 | KNOTTED1-like homeobox gene 4 | TALE | *KNAT4* |
| BnaAnng37140D | AT2G24830 | Zinc finger (CCCH-type) family protein / D111/G-patch domain-containing protein | C3H | *CCCH-type* |
| BnaC03g63840D | AT4G20380 | LSD1 zinc finger family protein | LSD | *LSD1* |
| BnaCnng29120D | AT3G01140 | Myb domain protein 106 | MYB | *MYB* |
| BnaAnng34960D | AT3G09230 | Myb domain protein 1 | MYB | *MYB1* |
| BnaC01g38230D | AT3G12820 | Myb domain protein 10 | MYB | *AtMYB10* |
| BnaA09g02150D | AT3G27810 | Myb domain protein 21 | MYB | *MYB1* |
| BnaAnng38560D | AT4G33450 | Myb domain protein 69 | MYB | *MYB 69* |
| BnaAnng13960D | AT5G01200 | Duplicated homeodomain-like superfamily protein | MYB | *MYB protein* |
| BnaC05g00840D | AT1G01060 | Homeodomain-like superfamily protein | MYB_related | *MYB_related* |
| BnaA09g00080D | AT4G01280 | Homeodomain-like superfamily protein | MYB_related | *MYB_related* |
| BnaCnng62330D | AT5G59780 | Myb domain protein 59 | MYB_related | *MYB59-2* |

ATO; *Arabidopsis thaliana* ortholog.
